# Human microRNA similarity in breast cancer

**DOI:** 10.1042/BSR20211123

**Published:** 2021-10-19

**Authors:** Ying Jing, Donghai Li

**Affiliations:** State Key Laboratory of Pharmaceutical Biotechnology, Jiangsu Engineering Research Center for MicroRNA Biology and Biotechnology, Nanjing Advanced Institute for Life Sciences (NAILS), School of Life Sciences, Nanjing University, Jiangsu 210023, P.R. China

**Keywords:** adipose tissue, breast cancer, disease, microRNA, similarity network

## Abstract

MicroRNAs (miRNAs) play important roles in a variety of human diseases, including breast cancer. A number of miRNAs are up- and down-regulated in breast cancer. However, little is known about miRNA similarity and similarity network in breast cancer. Here, a collection of 272 breast cancer-associated miRNA precursors (pre-miRNAs) were utilized to calculate similarities of sequences, target genes, pathways and functions and construct a combined similarity network. Well-characterized miRNAs and their similarity network were highlighted. Interestingly, miRNA sequence-dependent similarity networks were not identified in spite of sequence–target gene association. Similarity networks with minimum and maximum number of miRNAs originate from pathway and mature sequence, respectively. The breast cancer-associated miRNAs were divided into seven functional classes (classes I–VII) followed by disease enrichment analysis and novel miRNA-based disease similarities were found. The finding would provide insight into miRNA similarity, similarity network and disease heterogeneity in breast cancer.

## Introduction

MicroRNAs (miRNAs) are endogenous non-coding RNAs with ∼22 nucleotides in length and inhibit target genes at the post-transcriptional level. A total of 1917 human miRNA precursors (pre-miRNAs) and 2654 mature miRNAs are available in miRBase sequence database [1]. Many human miRNAs have been recognized as gene regulators in physiological and pathophysiological conditions.

To rapidly identify and characterize novel miRNA function, network analysis is increasingly emerging. Several publications have studied miRNA functional similarity, in which Gene Ontology (GO)-based methods (iRGOFS, MFS_GO), consensus-based network inference method (miRsig), miRNA target site accessibility and topology of target gene network-based method, miRNA-disease association-based method (MISIM), graph theoretic property-based method (miRFunSim), protein–protein interaction (PPI) network-based method in combination with GO and graph theoretic properties (PPImiRFS) have been developed [2–8]. MiRNAs have a pleiotropic effect and synergistically inhibit their target genes. MiRNA synergistic networks have been constructed based on target genes, GO, Kyoto Encyclopedia of Genes and Genomes (KEGG) pathway, PPI network and transcriptional regulation [9–12]. These methods and networks could shed light on function and synergism of miRNAs.

Breast cancer is a highly heterogeneous disease and the most common cancer in women worldwide. There are four major breast cancer subtypes: HR^+^/HER2^−^ (‘Luminal A’), HR^−^/HER2^−^ (‘Triple Negative’), HR^+^/HER2^+^ (‘Luminal B’) and HR^−^/HER2^+^ (‘HER2-enriched’). MiRNAs serve as oncogenes or tumor suppressor genes, and expression patterns alter across breast cancer subtypes and stages. One of network studies has constructed miRNA–miRNA functional synergistic network and identified key synergistic miRNAs and potentially stronger synergistic effect of miRNA family in breast cancer [9]. Additionally, functional synergistic miRNA–miRNA networks across diverse cancer types including breast invasive cancer have been reported [11]. Apparently, a comprehensive miRNA similarity network could further improve current knowledge.

MiRNAs function in signaling pathway and diseases through binding to their target genes by sequence complementarity. MiRNA diversity and cancer heterogeneity facilitate complexity of miRNA function and miRNA–disease association. In the present study, a combined similarity network was constructed based on mature sequences, target genes, pathways and functions and enabled a classification of breast cancer-associated miRNAs.

## Methods

### Breast cancer-associated miRNA data sources

Regulatory miRNAs of interest were extracted from miRCancer and PubMed [13–39]. A total of 264 miRNA entries were extracted from miRCancer database, and 108 miRNAs were obtained from PubMed database.

### Construction of miRNA similarity networks

miRPathDB calculated similarity scores of miRNA mature sequence, target genes or pathways [40]. Briefly, Hamming distance and Jaccard coefficient were calculated to define similarity of sequence, predicted target and target pathway, respectively. TargetScan, miRTarBase and MiRanda predicted target genes of miRNAs [41–43].

MISIM web server calculated miRNA functional similarity based on positive and negative miRNA–disease associations [44]. ‘ALL vs ALL Similarity’ option calculated paired functional similarity values for miRNAs. For all similarity analyses, threshold is 0.5.

Similarity networks was visualized, merged and analyzed by Cytoscape [45]. Venn diagram was done by FunRich [46].

### Disease enrichment analysis

miEAA 2.0 performed enrichment analysis (analysis method: over-representation analysis, adjusted *P*-value <0.05) [47]. Represented were top five diseases plus breast cancer according to adjusted *P*-values.

## Results

### Collection of breast cancer-associated miRNAs

A total of 372 miRNA entries were evidenced by experimental data related to breast cancer. PubMed and miRCancer were the key miRNA data sources. Regardless of miRNA expression, the extraction had only one criterion – miRNA of interest regulated breast cancer, as demonstrated by the experiments. Notably, miRNA expression was considered for subsequent functional similarity analysis. Some reviews have summarized breast cancer-associated miRNAs which have been covered by the present miRNA list. Fifty predicted breast neoplasm-associated miRNA were found using heterogeneous information network with GraRep embedding model, and half of them almost overlapped with the present miRNA list [48]. Together, this suggested that breast cancer-associated miRNAs were successfully collected.

To calculate similarity score and construct similarity network, specially functional similarity network, the miRNA list was further screened and narrowed down to 272 pre-miRNAs. Well-characterized cancer-associated miRNAs include miR-17-92 cluster (oncogene/tumor suppressor gene), miR-21 (oncogene), miR-221/222 (oncogene), let-7 (tumor suppressor gene), miR-15/16 (tumor suppressor gene), miR-200 (tumor suppressor gene) and miR-34 (tumor suppressor gene). To simplify and optimize similarity networks, these miRNA-associated networks were emphasized and analyzed [49].

### Sequence, target gene, pathway and function similarities and similarity networks

**Figure 1:**
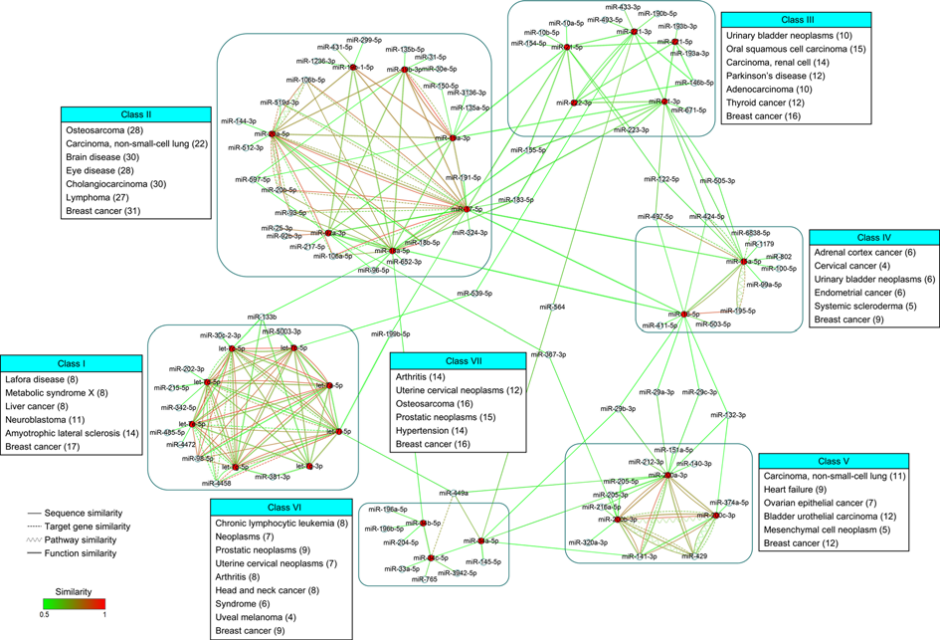
Similarity network, classification and enrichment analsysis of breast cancer-associated miRNAs Emphasized cancer-associated miRNAs are displayed in red. Values in parentheses represent numbers of observed miRNAs in relevant diseases.

Mature sequence of miRNA is of importance to regulate its targets through sequence complementarity of target mRNA 3′-UTR. Therefore, mature sequence was used to calculate sequence similarity score based on Hamming distance method. As shown in Figure 1, sequence similarity network is the largest one comprising four subnetworks and 102 miRNAs.

Target genes were predicted by three target prediction servers and utilized to calculate similarity scores of target genes and target pathways. Similarity networks of target genes and pathways are composed of seven subnetworks (27 miRNAs) and three subnetworks (8 miRNAs), respectively. The smallest similarity network is pathway-associated.

Functional similarity network was constructed based on miRNA–disease associations. Forty-seven miRNAs formed five subnetworks by similarity score. Besides sequence similarity, these miRNAs mainly showed certain functional similarity.

Although well-characterized miRNAs were emphasized and their similarity networks were extracted, only five shared miRNAs (miR-15a-5p, miR-200b-3p, miR-200c-3p, miR-20a-5p, miR-429) were found in similarity network of sequences, target genes, pathways and functions (Supplementary Figure S1). Otherwise, sequence, target gene and pathway similarity networks shared three miRNAs; sequence, target gene and function similarity networks consisted of 11 shared miRNAs. Of these shared miRNAs, five miRNAs (miR-20b-5p, miR-106b-5p, miR-141-3p, miR-195-5p, miR-429) did not belong to above-mentioned emphasized miRNAs but contributed to cancer-associated KEGG pathways, including pathways in cancer, miRNA in cancer and cell cycle (Supplementary Table S1, miRPathDB, *P*<0.05).

### Similarity network-based classification and disease enrichment analysis

To clearly annotate breast cancer-associated miRNAs, the combined similarity network was artificially classified into seven functional classes (classes I–VII) based on edge density and similarity (Figure 1). The classes contain the following emphasized miRNAs: let-7a-5p, let-7b-5p, let-7c-5p, let-7d-5p, let-7e-5p, let-7g-5p, let-7g-3p, let-7i-5p (class I), miR-17-5p, miR-18a-5p, miR-19a-3p, miR-19b-3p, miR-19b-1-5p, miR-20a-5p, miR-92a-3p (class II), miR-21-5p, miR-21-3p, miR-221-5p, miR-221-3p, miR-222-3p (class III), miR-15a-5p, miR-16-5p (class IV), miR-200a-3p, miR-200b-3p, miR-200c-3p (class V) and miR-34a-5p, miR-34b-5p, miR-34c-5p (class VI). Exceptionally, miRNAs in class VII are devoid of emphasized miRNAs and become connectors between other classes. Key genes in significant pathways of breast cancer subtypes and stages were used to inversely predict their regulatory miRNAs [50]. Surprisingly, partial miRNA connectors in class VII were related to breast cancer subtypes and stages (data not shown).

Disease enrichment analysis of miRNAs in classes I–VII showed that most miRNAs in all classes were enriched in breast cancer, and other diseases in classes I–VII were mainly implicated in cancers. Top 1 non-cancer diseases (Lafora disease, arthritis) were found in classes I and VII. Disease–symptoms network analysis has demonstrated that there is expected similarity between Lafora disease/arthritis and cancer based on symptom similarity and protein interaction [51]. Lafora disease is a neurodegenerative disease. Neuronatin aggregation within cortical neurons is a hallmark of Lafora disease. MiR-708, a member of 272 pre-miRNAs, has been reported to target neuronatin and regulate metastatic breast cancer [52]. Arthritis is a chronic condition involved in joint pain or joint disease. A potential arthritis–breast cancer association is that arthritis drugs could prevent breast cancer bone metastasis [53].

## Discussion

MiRNA similarity network is useful for functional annotation of novel miRNA, miRNA–disease association and classification in a unique disease. To better recognize similarity network in single cancer, a combined similarity network was constructed based on mature sequences, target genes, pathways and functions of breast cancer-associated miRNAs. Classification and enrichment analysis of these miRNAs were further performed.

Target prediction algorithms of miRNAs primarily pay attention to miRNA mature sequences, especially seed sequences. These miRNA of interest mostly showed the similarity of mature sequences establishing a maximum similarity network. However, the sequence similarity did not lead to identical similarity of target genes, pathways and functions. An possible explanation for this is that different similarity algorithm may result in inconsistent similarity scores. For functional analysis of miRNAs, miRNA–disease association is successfully used to calculate functional similarity of miRNAs. To date, several publications have proposed miRNA–disease association methods, including combinatorial prioritization-based method, network similarity integration method, multiple features-based method, network-based inference method and its combinational method with disease phenotypic similarity-based inference [54–56]. In non-model species without disease database, miRNA similarity method has been also developed [8]. To minimize the biases and represent a high-quality similarity network, similarity comparisons of mature sequences, target genes, pathways and functions of breast cancer-associated miRNAs were performed and applied for constructing a combined similarity network. Combination of four independent subnetworks provided an overview of miRNA similarity network and highlighted the relationships between individual similarity networks.

Network analysis is able to investigate molecular and biomedical networks, including PPI network, function linkage network, transcriptional regulatory network, metabolic network, phenotype network and drug–target network. In 2015, a knowledge-based computational framework ‘network fingerprint’ was introduced to analyze biomedical networks [57]. Disease network analysis identified disease–disease and disease–signaling pathway associations. As published previously, a combinational non-miRNA method of semantic and gene function association (SemFunSim) has been put forward and compared with other multiple methods [58]. A transcriptomics and epigenomics data driven approach has identified disease similarity at the molecular level [59]. A biological network (disease–miRNA, miRNA–gene, disease–gene and protein–protein interactome)-based method (mpDisNet) has been established to infer disease–disease relationship [60]. As shown in Figure 1, enrichment analyses of classes I–VII miRNAs pointed out possible miRNA-based disease similarity. Previous studies on disease similarity are partially consistent with the present miRNA-based disease similarity such as breast cancer-associated similarities of adenocarcinoma, adrenal cortex neoplasms, amyotrophic lateral sclerosis, arthritis, brain disease, carcinoma (non-small-cell lung), carcinoma (renal cell), cholangiocarcinoma, endometrial neoplasms, head and neck neoplasms, heart failure, hypertension, leukemia (lymphocytic), liver neoplasms, lymphoma, melanoma, metabolic syndrome X, neoplasms, neuroblastoma, osteosarcoma, ovarian neoplasmas, Parkinson’s disease, prostatic neoplasms, systemic scleroderma, thyroid neoplasms, urinary bladder neoplasms, uterine cervical neoplasms and various syndromes [51,58,60]. Although eye disease was found in class II, similarity between breast cancer and eye disease had been not definitely demonstrated. Twenty-eight breast cancer-associated miRNAs in class II contributed to eye disease enrichment and were also found in ncRNAomics-eye disease database Nc2Eye [61]. Most miRNAs in class II have a role in age-related macular degeneration. Another supporting result was from mpDisNet analysis, which showed breast cancer-associated similarities of cataract, diabetic retinopathy, glaucoma, Graves’ disease and retinoblastoma. Other cancers are class III-associated oral squamous cell carcinoma, class IV-associated cervical cancer and class V-associated bladder urothelial carcinoma, mesenchymal cell neoplasm and ovarian epithelial cancer. Otherwise, classes I–VII are not independent and linked by similarity of sequence and function derived from miRNA–disease association. Functional similarity is a unique linker between classes II and III, and classes II and VI, suggesting that miRNAs in classes II–VI have disease-based functional similarity and may co-regulate diseases.

Significance of miRNAs as diagnostic, prognostic biomarkers and therapeutic targets to clinic has been evaluated in basic research or clinical trial [14]. Partial miRNAs in each miRNA class are potential diagnostic, prognostic biomarkers. MiRNA signatures are essential for stage and subtype identifications in breast cancer [62–65]. MiRNAs in classes II–V and classes I–IV, VII contributed to stage and subtype of breast cancer, respectively. In contrast with predicted result, few experimental data have shown that miRNA connectors in class VII significantly refer to stage and subtype in breast cancer. Oncogenes and tumor suppressor genes could be therapeutic targets. These potentially therapeutic targets are distributed across all classes. Oncology network analysis would help to understand molecular mechanism underlying tumorigenesis and cancer progression. Different from similarity network analysis, a study focused on the change in miRNA–target regulation across subtypes in non-small cell lung cancer to construct a miRNA dysregulational synergistic network, discover miRNA dysregulatory modules and select potential biomarker [66]. For a combined similarity network, mapping of miRNAs with novel physiological and pathophysiolgical roles could infer molecular mechanism underlying breast cancer and potential application for biomarker and therapeutic target based on well-characterized breast cancer-associated miRNAs. Apparently, functional complementarity or redundancy of miRNAs in classes I–VII would influence treatment of breast cancer. In-depth experiments are also needed to support the similarity network and elucidate molecular mechanisms of breast cancer.

Brown adipose tissue (BAT) with a hallmark of mitochondrial uncoupling protein 1 acts as a key therapeutical target to mitigate obesity via energy expenditure. It is suggested that white adipose tissue (WAT) could enhance thermogenesis by the process of ‘browning’. MiRNAs also modulate brite/brown adipogenesis [67], intriguingly, in which regulatory miRNAs are almost encompassed in breast cancer-associated miRNA list. It suggests that adipose tissue regulation and breast cancer have specific similarity at regulatory miRNA level. Also, obesity is one of risk factors for breast cancer and menopausal status, and breast cancer is a factor associated with higher incidence of BAT activation.

In summary, a combined similarity network was constructed to elucidate similarity of sequences, target genes, pathways and functions of breast cancer-associated miRNAs. The similarity network analysis may promote the understanding of sequence, target gene, pathway, function and disease associations of miRNAs.

## Supplementary Material

Supplementary Figure S1 and Table S1

## Data Availability

The detailed methods and datasets used and/or analyzed during the current study are available from the corresponding author on reasonable request.

## Abbreviations

BAT: brown adipose tissue
GO: Gene Ontology
KEGG: Kyoto Encyclopedia of Genes and Genomes
miRNA: microRNA
PPI: protein–protein interaction
pre-miRNA: microRNA precursor
WAT: white adipose tissue
