# Supplementary material for: Human microRNA similarity in breast cancer
Source: Biosci Rep. 2021 Oct 19;41(10):BSR20211123. doi: 10.1042/BSR20211123 (PMC8529337; doi:10.1042/BSR20211123)
Supplement: Supplementary Figure S1 and Table S1 [file BSR-2021-1123_supp.pdf]

Supplementary Data

Human microRNA similarity in breast cancer

Ying Jing[1], Donghai Li[1*]

[1]State Key Laboratory of Pharmaceutical Biotechnology, Jiangsu Engineering Research Center for MicroRNA Biology and Biotechnology, Nanjing Advanced Institute for Life Sciences (NAILS), School of Life Sciences, Nanjing University, Jiangsu 210023, P.R. China.

*** Correspondence:**

Donghai Li

State Key Laboratory of Pharmaceutical Biotechnology, Jiangsu Engineering Research Center for MicroRNA Biology and Biotechnology, Nanjing Advanced Institute for Life Sciences (NAILS), School of Life Sciences, Nanjing University, Jiangsu 210023, P.R. China

E-mail: donghaili@nju.edu.cn

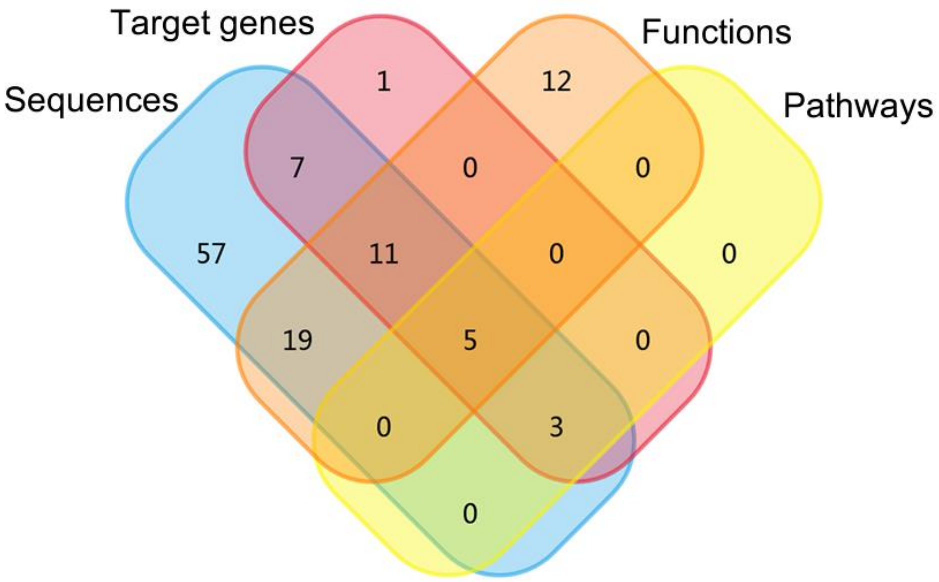

Supplementary Figure S1. Venn diagram comparing the miRNAs related to sequences, target genes, pathways and functions in similarity networks.

Supplementary Table S1. Pathway analysis of hsa-miR-20b-5p, hsa-miR-106b-5p, hsa-miR-141-3p, hsa-miR-195-5p and hsa-miR-429 (miRPathDB, *p*<0.05).

MiRNAs	Pathway	*p*-value	Targets
**hsa-miR-20b-5p**	Pathways in cancer	5.00E-04	*AKT3, CDKN1A, FZD6, HIF1A, MAPK9, PPARG, PTEN, STAT3, VEGFA*
**hsa-miR-106b-5p**	Cell cycle	4.38E-05	*CCND1, CCND2, CDKN1A, E2F1, E2F5, RB1, RBL1, RBL2, WEE1*
**hsa-miR-141-3p**	MiRNAs in cancer	2.82E-06	*CDC25A, CDC25C, CYP1B1, E2F3, MCL1, MDM4, PTEN, SHC1, TGFB2, ZEB1, ZEB2, ZFPM2*
**hsa-miR-195-5p**	Pathways in cancer	3.99E-07	*ABL1, AKT2, APC2, AR, ARAF, ARNT, ARNT2, AXIN2, BCL2, BCL2L1, BCR, BID, BIRC5, BMP2, CASP3, CASP9, CBL, CBLB, CBLC, CCDC6, CCND1, CCNE1, CCNE2, CDC42, CDH1, CDK4, CDK6, CDKN1A, CDKN2B, CHUK, CKS1B, COL4A1, COL4A3, COL4A4, CRK, CRKL, CTBP2, CTNNA1, CTNNA3, CXCL8, CYCS, DAPK3, DCC, DVL1, DVL2, DVL3, E2F1, E2F2, E2F3, EGFR, EGLN1, EGLN3, ELOB, ELOC, FGF1, FGF10, FGF11, FGF14, FGF18, FGF19, FGF2, FGF20, FGF23, FGF5, FGF8, FGF9, FGFR1, FGFR2, FGFR3, FLT3, FOS, FOXO1, FZD10, FZD3, FZD4, FZD6, FZD7, FZD8, FZD9, GLI2, GLI3, GSK3B, HDAC2, HHIP, HIF1A, HRAS, HSP90AA1, HSP90B1, IGF1, ITGA2, ITGB1, JAK1, JUP, KIT, KLK3, KRAS, LAMA4, LAMC1, LAMC3, LEF1, MAP2K1, MAP2K2, MAPK1, MAPK10, MAPK3, MAPK8, MAPK9, MAX, MECOM, MITF, MSH2, MSH3, MSH6, NFKB1, NKX3-1, NOS2, PAX8, PDGFA, PDGFRA, PDGFRB, PIAS2, PIK3CA, PIK3CB, PIK3CG, PIK3R1, PIK3R2, PIK3R3, PIK3R5, PLCG1, PLD1, PML, PPARD, PPARG, PRKCA, PRKCG, PTCH1, PTCH2, PTEN, PTGS2, RAC2, RAF1, RALB, RALBP1, RALGDS, RARB, RASSF1, RBX1, RET, RHOA, RUNX1, RUNX1T1, RXRA, SHH, SMAD2, SMAD3, SMO, SOS2, STAT1, STAT3, STAT5A, STAT5B, STK36, SUFU, TCF7, TCF7L2, TFG, TGFA, TGFB2, TGFBR1, TGFBR2, TP53, TPM3, TRAF1, TRAF2, TRAF3, TRAF4, TRAF5, TRAF6, VEGFA, VEGFB, VEGFD, WNT2B, WNT3, WNT3A, WNT4, WNT5B, WNT7A, WNT7B, WNT8A, WNT8B, WNT9A, WNT9B, XIAP, ZBTB16*
**hsa-miR-429**	MiRNAs in cancer	8.22E-15	*BCL2, BCL2L11, CDKN1B, CRKL, DDIT4, DNMT1, EP300, EZH2, FSCN1, IRS1, KRAS, MAPK7, MYC, PLCG1, PTEN, SHC1, VEGFA, ZEB1, ZEB2, ZFPM2*
